# The Challenges of Nursing Presence at the Patient's Bedside from the Perspective of Nurses: A Qualitative Study

**DOI:** 10.4314/ejhs.v33i2.13

**Published:** 2023-03

**Authors:** Tahereh Fallahnezhad, Bahman Aghaie, Reza Norouzadeh, Abbas Ebadi, Mohammad Abbasinia

**Affiliations:** 1 Medical-Surgical Nursing, Alborz University of Medical Sciences, Alborz, Iran; 2 Department of Medical-Surgical Nursing, Faculty of Nursing, Qom University of Medical Sciences, Qom, Iran; 3 Nursing and Midwifery Faculty, Shahed University, Tehran, Iran; 4 Behavioral Sciences Research Center, Life style institute, Nursing Faculty, Baqiyatallah University of Medical Sciences, Tehran, Iran; 5 Department of Medical-Surgical Nursing, Faculty of Nursing, Qom University of Medical Sciences, Qom, Iran

**Keywords:** Nursing presence, Challenges, Patient, Content analysis

## Abstract

**Background:**

Presence is one of the vital aspects of nursing care that improves the outcome of treatment, self-care, satisfaction, loneliness, and anxiety of patients. The literature review shows that most of the studies have been conducted on the conceptual analysis of the presence of nurses, while there are many challenges for the presence of nurses at the bedside. Therefore, this study aimed to determine the perceived challenges of nurses from being at the bedside of patients.

**Methods:**

This is a qualitative descriptive study. Conventional inductive content analysis was used. Field notes and in-depth semi-structured interviews were conducted with nineteen clinical nurses. Participants were selected from general and intensive care units. Data analysis was performed using Zhang and Wildemuth approach.

**Results:**

Three categories emerged: (1) professional challenges with subcategories: Supervision of novice nurses, insufficient skills and cultural unfamiliarity, (2) Management challenges with subcategories: managers' negligence toward nurse's needs, Strategies of nursing managers, lack of medical staff, inadequate medical facilities, inappropriate planned visits and appointments, (3) work environment challenges with sub-categories: ward overcrowding and non-standard hospital environment.

**Conclusions:**

This study identified the challenges and obstacles of nurses' presence at the bedside in different dimensions from the perspective of clinical nurses. To increase the presence of nurses at the bedside, nursing managers should create a standardized work environment.

## Introduction

Nurse presence is an important component of care (1). The presence of the nurse is defined as a therapeutic value for the nurse when interacting with the patients (2). Characteristics of nurse presence include being unique, connecting to the patient experience, sensing, going beyond scientific evidence, and being with the patient (3). In a meta-synthesis of the concept of presence in nursing, nurses should have personal maturity, morality, leadership commitment and respect for differences amongpatients (4). Patient and family satisfaction is a primary professional goal of nursing (5). Nurses ensure patient satisfaction through comprehensive care at the bedside(6). Practical nursing research shows that nurses must be clinically competent to be passionately present at the bedside (7). Mojarad et al showed that nurses with the skills of creating friendship, kindness and professional commitment can be more effective at the bedside (8). It has been shown that a quality nursing practice requires a balance between knowledge, presence and care. In particular, the presence of the nurse includes the sensitive, empathic and humanistic aspects of client care that enable a safe therapeutic relationship between the nurse and the patient (9,10). Hospitals that have a supportive environment for the presence of nurses at the bedside can reduce the feeling of loneliness and anxiety of patients and improve the treatment, care, and satisfaction of patients (6, 9,11). In addition, communication, balance, healing, growth and excellence are known outcomes of nursing presence(12). Gyeong et al found that a nurse presence program reduces stress and increases coping ability in the elderly (13). On the other hand, the insufficient nurse presence brings negative consequences such as patient dissatisfaction and poor communication with patients (14,15).

One of the goals of health managers is the efficient presence of nurses in complex care environments to achieve positive results in patients and medical centers (6, 16). Our research has revealed that nurses' perspectives on nurse presence have not been investigated in Iran. Molazem et al showed that the presence of a nurse helps the patient's psychological security and meeting information needs (17). Holistic nursing care and creating a sense of physical, mental and spiritual well-being in patients requires an effective nursing presence (18). Learning the concept of presence can potentially enhance nursing care (19). Nurses need to understand the nature and dimensions of nurse presence in order to perform professional roles, such as assessing patient needs (4, 20). Yen et al shows that nurses spend most of their time in patients' rooms, but nurses are often unaware of the results of their presence (16). Despite the research conducted in this field, the barriers and facilitating factors for the presence of nurses at the bedside have not been identified (21). Increasing patient care time is an important goal of health managers. This leads to more presence of nurses at the bedside. Despite efforts to increase care time through the provision of electronic systems, this has not yet been achieved (22, 23).

The literature review shows that most of the studies are focused on the concept of nursing presence (24–27), patients' perception of the nursing presence (28), and nurses' knowledge of the importance of nursing presence. However, the body of knowledge about the concept of presence and its dimensions does not provide an explanation for the challenges of nursing presence at the patient's bedside. Increasing the length of time and improving the quality of nursing presence requires identifying the obstacles and support conditions that nurses face in the field. Also, Welch et al said that the absence of a nurse makes the nurse unaware of the patient's needs and this puts the patient at risk (29).

Considering the importance of nurses' presence in the nursing profession, this qualitative study was undertaken to explain the perceived challenges of nurses from being at the bedside of patients. By identifying the challenges of nurses in this field, solutions can be proposed according to their professional role.

## Materials and Methods

This study is a qualitative descriptive study using the conventional inductive content analysis method. The content analysis method is useful when sufficient knowledge of a phenomenon is not available (30). Therefore, this research method was used due to the lack of sufficient knowledge of the challenges of nurses' presence at the bedside.

**Setting and participants**: Nurses with a maximum variance were selected (Table 1). Nineteen nurses from the general and intensive care units (teaching and private hospitals of Alborz province, Iran, 2021) were selected purposefully. The inclusion criteria were to have at least a bachelor's degree in nursing and one year of clinical experience.

**Table 1:** Demographic characteristics of participants

Participant Number	Age	Gender	Work setting	Educational Level	Clinical Experience (Year)
1	36	Female	General	Bachelor's degree	12
2	33	Male	ICS	Bachelor's degree	6
3	41	Female	General	Bachelor's degree	19
4	44	Female	General	Bachelor's degree	18
5	26	Female	General	Bachelor's degree	3
6	39	Male	General	Bachelor's degree	14
7	25	Female	General	Bachelor's degree	2
8	31	Male	General	Master's degree	3
9	33	Female	ICS	Master's degree	7
10	43	Male	General	Bachelor's degree	20
11	29	Female	General	Bachelor's degree	5
12	38	Female	ICS	Master's degree	10
13	39	Male	ICS	Bachelor's degree	14
14	36	Female	General	Bachelor's degree	10
15	33	Male	General	Bachelor's degree	9
16	39	Female	ICS	Master's degree	13
17	47	Female	General	Bachelor's degree	20
18	29	Female	ICS	Bachelor's degree	6
19	50	Male	General	Bachelor's degree	26

Intensive care settings: ICS

**Data collection**: Data collection was done through semi-structured in-depth interviews and field notes. The time and place of the interviews were agreed with the participants. The interview guide was prepared based on the opinions of the research team members, with the consultation of a qualitative research expert outside the study group, and two pilot interviews. Interviews conducted by the corresponding author in Persian. The corresponding author is a qualitative research specialist and a nursing educator. After presenting the objectives of the study to the participants, the interview began with an open-ended question: “Tell me about your experience at the patient's bedside.” The follow-up question included “What barriers do you face to be at the bedside?” Each interview lasted for 30–60 min. The categories and subcategories were reviewed continuously by the research team. Data saturation was achieved in the 17th interview. Two additional interviews were performed to ensure that no new data emerged. Field notes were prepared by the corresponding author. Interviews were audio-taped and fully transcribed verbatim in Microsoft Office (ver., 2007) on the same day.

**Data analysis**: Data analysis was performed simultaneously with data collection using Zhang and Wildemuth approach (31) Data analysis steps include: (1) The recorded interviews were transcribed word for word after each interview on the same day, (2) semantic units were extracted and coded from the sentences and words and reviewed by the authors of the article, (3) After three sessions of peer review, the codes were classified into five sub-categories and three categories based on their similarities and differences, (4) The clarity and consistency of the extracted codes were checked to correct the subcategories, (5) data collection and analysis until saturation, (6) Iterative verification of accuracy and consistency of data by the authors, two nurses and peer reviewers (7) finalization of categories and sub-categories, and (8) reporting on all phases of the study.

**Trustworthiness**: According to Lincoln and Guba, credibility was established by allocating enough time for each interview, prolonged engagement of the research team, and member checks by eight participants. A single interview was conducted with the participants during each session. Follow-up interviews were conducted after completion and analysis of the previous interview. All interviews were checked by participants. For confirmability, throughout the study, two external reviewers with qualitative research skills checked the quality of the interviews, coding, and categories to reach consensus. For dependability, all stages of the study were reported in detail. Finally, transferability was established through maximum variation sampling in age, gender, clinical experience, type of hospital and ward, work position, and level of education (32,33).

**Ethical considerations**: The Ethics Committee of Alborz University of Medical Sciences approved this research (approval code: IR.ABZUMS.REC.1399.103). Verbal consent was obtained from the participants. The Ethics Committee at Alborz University of Medical Sciences approved the verbal consent for the study participation. Participants were informed about the study aim, voluntary participation, and the right to withdraw from the study. Permission was obtained from the participants for audio recording. The authors confirm that all methods were performed in compliance with relevant guidelines and regulations.

## Results

Nineteen nurses participated in the study. The average age of nurses was 38.61 ± 4.2. Fifteen had a bachelor's degree in nursing. 13 nurses worked in general wards (Table 1).

In total, 416 codes were extracted. The extracted codes were classified into thirteen initial subcategories. After the final analysis by the research team, the subcategories with semantic and conceptual similarity were merged and 10 subcategories were placed in three categories. Three categories include professional challenges with three sub-categories, management challenges with five subcategories, and work environment challenges with two subcategories (Table 2).

**Table 2:** The Categories and sub-categories

Example of coding	Sub-categories	Categories
Time-consuming inspection rounds accompanied by a supervisor, Unscheduled rounds of supervisors, Spend a lot of time helping and monitoring novice, Lack of professional skills in communicating with a patient, and Unfamiliarity with the culture and behavior of patients	Supervision of novice nurses	Professional Challenges
	Insufficient skills Cultural unfamiliarity	
Managers ignore the low salaries of nurses, Nurses' dissatisfaction with managers, Lack of motivation and energy due to fatigue and burnout of nurses, Having a heavy workload due to a shortage of nurse, Waste of time due to shortages of medical equipment, and Lack of specific time for morning doctor visits	Managers' negligence toward nurses' needs	Management Challenges
	Strategies for nursing managers Shortage of medical staff	
	Inadequate medical facilities Inappropriate planned visits and appointments	
Crowded ward due to the presence of many medical students in the ward, Inadequate conditions of patients' rooms	Ward overcrowding	Work Environment Challenges
	Non-standard hospital environment	

**Professional challenges**: The quotations show that human factors can reduce the time nurses are effectively present at the bedside. This category includes supervision of novice nurses, insufficient skills, and cultural unfamiliarity.

**Supervision of novice nurses**: Participants stated that supervising novice nurses takes a lot of time and reduces their presence at the patient's bedside.

*“We have to spend a lot of time supervising nursing students or novice nurses. This problem makes us not be able to spend more time at the patient's bedside” (P. 10).*

*“The nurse talked to the head nurse about the time-consuming supervision of a new nurse. The nurse said that it reduces her time at the bedside and the patients are upset about this situation...she asked head nurse to take this responsibility herself” (field note1).*

**Insufficient skills**: Participants reported poor patient communication skills, poor teamwork, and insufficient technical knowledge in nursing interventions that made them afraid to be at the bedside. Their fear makes them less likely to be at the bedside. This anxiety prevented nurses from improving their clinical skills. *“When nurses have poor skills, the nurse is afraid that the patient will notice this weakness” (P.3).*

**Cultural unfamiliarity**: The gender difference between the nurse and the patient and not being familiar with the patient's culture leads to disruption in the relationship between the nurse and the patient and job stress. “*This hospital is a referral hospital. Patients are admitted with different cultures and beliefs; Sometimes it is very stressful to be at the patient's bedside because you are not familiar with the patient's culture and beliefs” (P.8).*

“*The nurse was unable to communicate with the non-Farsi speaking male patient. She was confused and anxious and asked her colleagues for help ” (field note 2).*

**Managerial challenges**: There were experienced situations by nurses that undermined by the nurses' presence at the patient's bedside. In this way, the nurses feel dissatisfied, tired and exhausted. Managerial attenuation consists of five sub-categories: managers' negligence toward nurses' needs, strategies of nursing managers, and shortage of medical staff, inadequate medical facility, inappropriate planned visits and appointments.

*4.2.1 Managers' negligence toward nurses' needs*

Nurses stated that managers' negligence towards their welfare facilities such as insurance, nutrition, and restroom causes job dissatisfaction and reduces their job motivation to presence at the patient's bedside.

“*Nursing managers do not try to solve the problems of salaries, high workload and amenities that cause discomfort and discouragement of nurses” (P. 13).*

*“As an experienced nurse, I know that hospital administrators are not interested in the needs of nurses. Therefore, I do not have much motivation to be at the bedside of patients” (P.6).*

**Strategies for nursing managers**: Nurses showed that the management style plays an important role in the presence of nurses at the patient's bedside. Autocratic decisions, rotation of nurses between wards without considering clinical preference and skill, and the use of functional division of labor reduces nurses' motivation to be at the bedside.

*“Nursing managers do not consult with us... Many times I have been transferred to other departments as an assistant nurse. This upsets me” (P.9).*

*“A new nurse tells the head of the surgical ward that she is very upset and nervous about being transferred from the ICU to the surgical ward. She said she is not interested in the new ward” (field note 3).*

The nurses stated: being forced to do non-clinical work such as completing forms and accreditation documents and in-service training prevents them from being at the patient's bedside. *“The workload is high... Doing accreditation program doubled the problem... This made difficulties for presence at the patient's bedside” (P.4).*

*The increase in the number of hospitalizations has made it impossible for us to effectively present at the patient's bedside” (P.14*).

Managers' decisions to manually record medical information in the medical record such as vital signs and nursing reports, patient education and the discharge process waste a lot of time in each shift. In this regard, one of the nurses described her experience. *“Frequent recording of information in a patient's medical record, which is often manual, is very time-consuming. It takes a lot of time for us” (P.6).*

*“The head nurse of refers the objection of nurses to the time-consuming documentations to the supervisor” (field note 4).*

***Shortage of medical staff**:* Shortages of nurses, nursing assistants, and physicians pose significant obstacles to bedside nurse attendance. Nursing staff shortages hindered optimal nursing bed ratios. These conditions caused nurses to suffer from mental and physical exhaustion. So they couldn't spend enough time at the patients' bedside. Also, due to the shortage of resident doctors, nurses spend a lot of time for telephone orders.

“*We have a shortage of nurses and assistant nurses in every shift, and this is our concern in every shift... The shortage of nurses and nurses creates a high workload” (P.2).*

*4.2.4 Inadequate medical facilities*

Worn medical equipment and frequent breakdowns waste the time of nurses.

*“Failure of medical equipment always causes trouble... We have to constantly worry about these devices not working properly. For example, pressure gauges oxygen or central suction devices. This wastes a lot of time. Sometimes we forget the patient” (P.3).*

*“Due to the shortage of blood pressure devices and failure monitoring device, the nurses waited a long time in the treatment room for the blood pressure devices to be ready and repair monitoring device” (field note 5).*

**Improperly scheduled visits and rounds**: According to the participants, poor planning of doctor's visits and clinical nursing rounds is related to the reduction of presence at the bedside. Nurses lose their useful time for patients because of irregular doctors' visits or the intrusive presence of supervisors. Interruption in nursing programs leads to a waste of time and reduces the number and duration of the presence of nurses at the patient's bedside.

*“Physicians do not have a specific schedule for visiting patients; doctors come to the ward to visit patients, sometimes at the beginning of work and sometimes at the end of work shifts. This disrupts our plans to presence at the patient's bedside” (P.6).*

**Work Environment challenges**: A set of factors related to the workplace, along with other factors can create barriers to the nurses ‘presence at the patient’s bedside. The category of unsuitable environments indicates a combination of important environmental factors for nurses to have an effective and high nursing presence at the patient's bedside. This category consists of three sub-categories: inadequate medical facility, ward congestion, and nonstandard hospital environment.

**Ward overcrowding**: Nurses stated that the presence of medical students and families increased congestion in the wards. Also, answering students' questions and providing facilities for their practice takes many hours for nurses.

*“The presence of medical students, families, and physiotherapist, etc., causes the ward to become crowded I cannot find enough time to be a presence at the patient's bedside” (P.3).*

**Non-standard hospital environment**: This condition reduces the nurses' willingness to be presented at the patient's bedside. Non-standard hospital departments waste nurses' time. The lack of sufficient facilities in the patient room, including air conditioning and sufficient space for each patient, creates challenging conditions for nurses which reduces the desire of nurses to be at the patient's bedside. *“Some rooms do not have enough light and because of the lack of air conditioning, the unpleasant odor and do not have good conditions... Under these difficult circumstances, we cannot have an effective and much presence of nursing the patient's bedside” (P.19).*

*The nurse is very upset by the heat and the unpleasant smell of her patient's room and is finding a mask for herself... opens the window and door of the patients' room” (field note 6).*

## Discussion

The results of the study show that clinical nurses have serious challenges to be at the patient's bedside. These include professional, managerial and work environment challenges that negatively affect patient care. From the perception of nurses, these challenges create job stress. In line with this finding, previous studies show that high workload, absence of doctors, non-supportive managers, insufficient human resources and unfavorable interpersonal interactions are known job stressors for nurses (34, 35).

**Professional challenges:** The lack of up-to-date knowledge and insufficient professional competence lead to increased anxiety and stress of nurses at the bedside. Therefore, fear and anxiety about being presence at the patient's bedside are unpleasant experiences. Consistent with this finding, Mohammadipour et al. found clinical competence and self-actualization are the antecedents of nursing presence. Amiri et al. shows that cultural unfamiliarity in cross-cultural care is always one of the causes of occupational stress in nurses(36). Accordingly, Katsantoni et al. shows that work stress causes fatigue in nurses. The results of studies show reports that nurses are in an ongoing and snowballing process in relationships with needy individuals and are prone to fatigue (37, 38). The myriad consequences of fatigue included a reduction in patient safety, diminished judgment, and decision-making, decreased reaction time, loss of concentration, absenteeism, clinical errors, and reduced quality of interaction with colleagues and patients (37, 39). The findings show that most nurses perceive mental and physical fatigue as an obstacle to being sufficiently present at the patient's bedside. In this study, nurses' poor competency is a managerial challenge for clinical nurses. In this case, the lack of up-to-date managerial knowledge and insufficient clinical competency leads to increased nurse anxiety and stress in the patient's clinical presence. Therefore, fear and anxiety about being presence at the patient's bedside are unpleasant experiences (24).

**Managerial challenges:** Nurses' statements indicate nursing management strategies undermine nurses' effective presence at the patient's bedside. Nursing managers' policy for frequent rotations is often unpleasant for nurses. Adopting a high turnover approach without considering nurses' skills and willingness reduces nurses' motivation to presence at the patient's bedside. In this regard, the results of studies show that the decisions of nursing managers, such as high turnover rates among nurses, leading to the shortage, job dissatisfaction, and intention to leave (40,41). This problem and documentation duties reduce the presence of nurses at the bedside. In recent years, the implementation of accreditation programs approved by the Ministry of Health and the need to develop care policies for nurses have caused a lack of time to address patients' care needs further. Studies show that management styles have a relationship with job satisfaction and nurse retention (42–44). Nursing management behaviors, such as attention to autonomy and motivation, improve nurses' performance (45, 46). An organizational challenge for nursing presence at the patient's bedside is managers' neglect of nurses' wages. In line with this finding, Cummings et al. found salary is associated with nurses' job dissatisfaction and is important for good nurse outcomes, but it does not decrease the work environment's impact and staffing on nurse outcomes (47).

**Work environment challenges**: In terms of environmental challenges, shortages, and failure of medical equipment, overcrowded and non-standard wards are barriers for nurses to presence at the patient's bedside. Similarly, McHugh et al. suggest the busy environment of the wards (noise and traffic), unsuitable environmental conditions (improper ventilation, heating, cooling, and lighting), and admission of critically ill patients in the wards are serious barriers to successful nurse-patient communication (48). An environmental challenge is the congestion of nursing and medical students for internships and the lack of a balanced distribution of students according to the number of patients in each ward. In such a crowded environment, the nursing staff refrains from being at the patient's bedside to avoid causing more discomfort to the patient. In this regard, Choudhury et al. showed patients reported that they would feel comfortable in medical students' presence in clinical education. However, 63.7% of patients preferred one or two medical students to be presence during a consultation (49). According to the experiences of the authors of this article, nursing students do a significant part of patients' affairs in the internship. This makes the nursing staff feel that all the needs of the patients are fulfilled by the students. This is a vicious cycle for effective presence.

In conclusion, this study identified the perspective of clinical nurses regarding the challenge of nursing presence. Some challenges are structural, and some are self-made. Nursing managers can optimize the presence of nurses at the patient's bedside by modifying the management method, creating a standard work environment and improving clinical competence. This requires serious efforts by nursing officials and clinical nurses. The authors of this article suggest using nursing management methods to increase motivation, employing nurses based on their expertise, desire and experience, and using the case management method in patient care. In addition, strengthening the hospital's physical structures and aligning accreditation programs with the real needs of patients and nurses could improve the presence of nurses at the patient's bedside.
